# Guidelines for Inspection of Companion and Commercial Animal Establishments

**DOI:** 10.3389/fvets.2018.00151

**Published:** 2018-07-06

**Authors:** Clifford Warwick, Mike Jessop, Phillip Arena, Anthony Pilny, Catrina Steedman

**Affiliations:** ^1^Emergent Disease Foundation, Tonbridge, United Kingdom; ^2^Veterinary Expert, Swansea, United Kingdom; ^3^Pro-Vice Chancellor (Education) Department, Murdoch University, Mandurah, WA, Australia; ^4^Access Specialty Animal Hospital, Culver City, CA, United States

**Keywords:** guidelines, inspection, husbandry, animal, health, safety, companion, pet

## Abstract

Various establishments exist in which animals are held for a variety of reasons. Historically, the management and inspection of animals in commerce and in private keeping have involved a considerable degree of arbitrary evaluation based on the personal experience of the vendor, keeper, advisor, or inspector. Accordingly, relevant protocols and standards are subject to considerable variation. Relatedly, diversity of traded and privately kept species generates significant challenges for those responsible for facility management and inspection alike. Animal welfare and public health and safety are constant and major concerns that require objective methodologies to monitor and control. This report focuses on establishments concerned with the boarding, breeding, storage, vending or handover of animals intended for human “companions” or “pets”, and aims to provide universal objective information for essential husbandry, inspection protocols and an allied inspection assessment tool for scoring establishments.

## Introduction

In the UK alone, there are estimated to be ~5,000 dog breeding centers and 4,500 pet stores (1). At least 13,000 species across all classes, and including domesticated and non-domesticated (wild) animals, are kept for human purposes, mostly as “pets” (2–4). This diversity of species introduces significant inspection and husbandry problems, for example, difficulties in identifying species and their legality, risk to human health and safety, and biological needs. Welfare of captive animals (notably exotics) is frequently and inherently compromised by captive conditions, for example, inability to exhibit natural behavior such as climbing, flying, and burrowing or to roam freely over large distances, as well as imposed abnormal conditions such as transparent boundaries that are not accepted psychologically by some species (5–8). In addition, there are important animal and human health issues that require appreciable understanding of risk prevention and zoonotic disease control (9–14).

Historically, the management and inspection of animal establishments, both in commerce and in private keeping, have involved a considerable degree of arbitrary evaluation based on the personal experience of the vendor, keeper, advisor, and inspector. Relevant protocols and standards are subject to wide variation, both locally and internationally (15). In addition, obtaining objective expert-led and evidence-based material uncompromised by vested interests while combining both essential animal husbandry and inspection guidance is challenging. Highly limited and often regional guidance on animal husbandry and inspection of certain facilities is available [for example (16–31)], and although this information is helpful, not all existing guidance is similarly structured or cohesive, and this review may provide an opportunity to ameliorate this situation.

Relatedly, scientific reviews and allied provision of tools for assessing the suitability of species are also available (32–35). Nonetheless, generalized, internationally relevant, combined husbandry with inspection guidance that includes assessment tools is lacking. Animal welfare and public health and safety must be regarded as constant and major concerns that require objective methodologies to monitor and control.

This report focuses on establishments concerned with the boarding, storage, breeding, vending or handover of animals intended as human “companions” or “pets”, and aims to provide universal objective information for both inspection protocols and essential husbandry. In order to enhance objectivity for any inspection process, we have proposed a novel scoring system. Using this scoring system, a “traffic light,” “star rating” or simple 0–3 number ranking can be utilized to “flag” or grade the facility in a manner not dissimilar to that adopted for restaurants and other establishments.

## Methods

We conducted an online literature search using Google Scholar, EMBASE, MEDLINE, Royal Society of Medicine, VetMed Resource, and Murdoch University and used the following key terms: animal, establishment, inspection, guidelines, companion, pet, best practice, husbandry, boarding, breeding, storage, wholesale, retail, vending, enforcement, licensing; with a key objective being to identify items that fulfilled our “three-asset” resource target—namely resources that addressed guidance regarding animal husbandry, inspection, and provision of a relevant assessment tool to aid the guidance. Twenty three publications of *prima facie* relevance were identified and reviewed for content that included combined guidance on animal husbandry and facility inspection, together with provision of an allied inspection assessment tool. Fifteen articles and reports were considered to be cogent. None of the identified items achieved the “three-asset” resource target. Essentially, the most welfare generous recommendations were selected from any reviewed publication. In addition, a working consultation group of independent experts familiar with animal husbandry and inspection provided input and also conducted reviews of the present guidelines thereby allowing high-level impartial experiential contributions in all relevant areas. Accordingly, this review primarily utilized literature-based evidence, and secondarily expert-led, experiential-based information, particularly in respect of the specific guidance sections.

Our research and review found several areas, for example those of relevance to establishment layout and quarantine protocols, where significant information was unavailable. However, these areas are important aspects of animal husbandry. Therefore, in areas of such information deficiency we have drawn on group consensus, some of which arguably is also “common sense,” for example avoidance of unnecessary environmental disturbances and other rational approaches to management.

## Interpretation

Terms used in this document are not intended to offer legal definitions, but are descriptions intended to clarify relevance and focus for both animal establishment managers and inspectors. Accordingly, meanings of the terms used herein are as follows:

1. “Animal establishment”: any operation or premises including pet stores, boarding facilities, breeders, and rescue shelters or “sanctuaries” in which “companion” or “pet” animals are kept or intended to be kept for any period whether for commercial or other reasons, and that may be required to undergo inspection.
2. “Companion” or “pet”: any animal kept or intended for personal, pleasure, or ornamental use rather than for agricultural food, sport, public exhibition, or research and experimental purposes.
3. “Handover”: the passing of an animal from an establishment to a recipient, whether the recipient is another establishment or individual for any reason including sale, donation, and post-boarding release.

## Relevant laws, regulations, and duty of care

Laws and regulations vary considerably across different nations and regions, therefore, it is essential that users of these guidelines independently ascertain any relevant legal requirements. It is beyond the scope of this article to attempt to present and discuss international regulations, laws, and cultural issues. Our aim is that, regardless of whether or not legal and cultural issues are well defined in a region or country, these guidelines may become aspirational and assist at some level to augment any structure or intention to determine or improve relevant issues. Animal establishment inspectors and managers have a legal or moral “duty of care” toward animals and the public, and should take reasonable steps in all circumstances to meet the welfare needs of the animals and to safeguard public health and safety to the extent required by good (preferably best) practice (36). Whether or not specific legal provisions are in place in any area, we recommend that certain foundation principles for laws, regulations and protocols necessarily include set obligations.

For example, in respect of animal welfare, The UK Farm Animal Welfare Council established the Five Freedoms for animal well-being (37), which are used here along with some elaboration by both The Farm Animal Welfare Council (38) and the Royal Society for the Prevention of Cruelty to Animals (39):

Five Freedoms:

1. Freedom from hunger and thirst—by ready access to fresh water and a diet to maintain full health and vigor;
2. Freedom from discomfort—by providing an appropriate environment including shelter and a comfortable resting area;
3. Freedom from pain, injury or disease—by preventing them from getting ill or injured and by making sure animals are diagnosed and treated rapidly if they do;
4. Freedom to express normal behavior—by providing sufficient space, proper facilities and company of the animal's own kind (*added:* where appropriate);
5. Freedom from fear and distress—by ensuring conditions and treatment, which avoid mental suffering.

These criteria were further updated by the FAWC (40) to include providing animals with a good quality of life, and animals that did not have an overall good quality of life should be viewed as below the legal threshold. Public health and safety considerations are fundamental remits of responsible governance and do not need elaboration, save as to emphasize that disease prevention and control should be central, and comprehensively applied to any activity that involves animal vending, keeping, inspection, and enforcement (6, 41).

## General assessment

Animal establishments should provide conditions consistent with best practice for intrinsic animal welfare reasons, but also because prospective acquirers of animals may regard the conditions in which livestock are kept and observed as examples demonstrating appropriate long-term care (6). Also, animals in temporary facilities may actually reside or remain there for relatively long periods, and therefore they ought to benefit from conditions that are appropriate for long-term captives (42). Conditions for animals at establishments should be exemplary in aiming to provide observers or purchasers with practical solutions that are appropriate for long-term animal husbandry, and not merely provide overly basic “temporary-style” conditions (3).

## Establishment and management protocols

An establishment or facility may be assessed for its suitability whether or not animals are present. Therefore, the general facilities (including all enclosures, and management protocols) should be established for inspection (43).

### Layout

The layout of the establishment must be assessed with the primary principle being the welfare of the animals. The atmosphere should be calm and quiet. The ambient lighting should be soft. Included in the assessment should be the ease of access for monitoring of the animals and cleaning of facilities.

General positioning of cages should avoid undesirable potentially stressful situations where the presence of relevant predatory species may lead to stress in relevant prey species (20). Accordingly, prey should be protected from visual, auditory or olfactory stress associated with potential predatory species (20). For example, the positioning of feline species within sight of most avian or small mammal-rodent species should be avoided, which should also minimize olfactory perception.

Cage stacking should be such that it avoids the risk of cross contamination, for example, feces, food, water, or substrate dropping into a cage from above. Animals must not be situated “too low down” where they may experience fright and stress from visitors or passers-by (for example, on the floor) (44). Cages should not be positioned where they can be easily and accidentally knocked or kicked, for example, in walkways or near doorways (44). The entry and subsequent passage of sunlight through widows should also be considered in relation to cages and the possibility of light disturbance or overheating (45). Similarly, cages should not be placed in close vicinity of heating elements or in the direct pathway of drafts to avoid these potential stressors (45).

### Staff

Staff should be sufficient in number and adequately qualified or experienced to maintain good supervision of both the premises and all animals (46). Where qualifications are required by law there should be evidence that the required pass level has been achieved. The holder of the qualification should be in a senior position at the establishment. Evidence of training protocols and details of training course attendance must be available. General knowledge should be checked at any inspection as well as evidence to convey that it is used effectively (20). Suitably trained and competent staff must be in charge of the animals at all times (20, 47), and be available 24/7 whether onsite or within nearby distance of the establishment. Deputization by senior staff to others must only be to suitably trained, experienced, and competent members of the team. Experienced staff must be available at all times whether or not the facility is open to the public. There should be evidence of staff rosters and contact details for hours outside of trading. There must be evidence that the animals are checked at least twice daily. Replacement and suitably trained staff should be available at short notice, should a key team member fall ill.

### Hygiene

#### Overall assessment

Inspection will assess the cleaning protocols and general hygiene conditions for the facility itself. This is separate and distinct from the individual cage cleaning and should be recognized as such by the facility.

The overall assessment will firstly consider general odor. This should be fresh and free from any obvious noxious or foul smell (20, 48). Equally it should be clear of an overpowering chemical odor where disinfectant or air freshener may have been used to mask poor hygiene. The general layout and tidiness of the facility should also be considered with an assessment of “cleanability” (20). If a facility is cluttered it will be difficult to clean effectively. An overview of the state of repair of the facility will also help with an assessment of general hygiene, for example poorly maintained or sealed flooring will significantly impair the ability to clean effectively.

In general, good cage hygiene—*cleanliness*—should be assessed by establishing the absence of foul or atypical odors, infrequency or absence of “smears” and other overt debris on walls and furnishings, absence of “compacted” and thus likely old and soiled substrata, absence of “pest” invertebrates such as mites, flies, fly larvae, and specific feeder invertebrates such as crickets where these are ubiquitous and/or free-running from cage to cage (20). Many pests may require very close inspection of furnishings, substrate and bedding to determine their presence. Water should always be clean and distinctly fresh.

The presence of small amounts of recent species-specific droppings are acceptable, and even recommended, as these may provide familiar chemical cues that animals find calming (49). However, this should not involve widespread soiling of the environment, but merely a discrete “chemical presence” within the enclosure.

#### Disease risk management: people

Precautions should include regular cleaning and good personal hygiene of staff in addition to effective quarantine of incoming groups of animals (50). Enclosures and relevant facilities should be disinfected with appropriate materials as necessary and always between different batches of animals. Disinfectant should be appropriate for the contaminants likely to be encountered as well as safe for the relevant species, which may involve other invertebrates being at risk of exposure to incidental insecticides (51). Staff should pay particularly close attention to hand cleansing between animal enclosures, to prevent cross contamination both of animals and cages, as well as the wider environment. Hand cleansing should include rigorous use of antibacterial soap and water to assist in removing primary debris, and a second cleansing tier using an alcohol-based antimicrobial agent (disinfectant hand gels alone should not be relied on) (14). If a towel is to be used for drying hands, disposable paper towels should be used in place of “tea towel” type materials because the latter are likely to harbor potential pathogens.

CAUTION: Hand washing offers very limited protection against microbial pathogens and their transmission from animal-to-animal, from animal-to-person, or from person to-person. All animals, cages, furnishings, staff (including clothes, hair), and the wider environment should always be considered potentially contaminated with both animal and human pathogens (14).

*Signage:* Clear signage should be in place cautioning the public with regards to zoonotic infections (52), and the heightened risk to children under five, pregnant women, the elderly, and immunocompromised from direct or indirect contact with animals and the shop environment (14).

Table 1 provides a short-list of common examples of animal-to-human (zoonotic) diseases that can be displayed in-facility to promote disease awareness.

**Table 1:** Zoonoses signs and symptoms.

**Zoonosis/condition**	**Source**	**Signs & symptoms**
Salmonellosis/gastroenteritis	Fish, amphibian, reptile, bird, mammal	Nausea, vomiting, diarrhea, abdominal cramps, and pain, fever, painful joints, meningitis, flu-like
*E. coli* infection/ gastroenteritis	Amphibian, reptile, bird, mammal	Nausea, vomiting, diarrhea, abdominal cramps, and pain, fever, painful joints, meningitis, flu-like
Campylobacteriosis/ gastroenteritis	Amphibian, reptile, bird, mammal-primate	Nausea, vomiting, diarrhea, abdominal cramps, and pain, fever, painful joints, meningitis, flu-like
Leptospirosis	Amphibian, reptile, bird, mammal	Flu-like, vomiting, icterus, telangiectasia, uveitis, splenomegaly, meningitis
Chlamydiosis	Bird, mammal-primate	Flu-like, pneumonia, fever, cough
Vibriosis	Fish, amphibian, reptile, bird	Gastrointestinal, pain, vomiting, fever, otitis
Lyme disease/bartonellosis	Mammal	Flu-like, fever, rash, gastrointestinal
Toxocariasis	Mammal	Eye problems
Giardiasis	Mammal-primate	Gastrointestinal, fever, nausea, fatigue, weight loss
Tuberculosis	Fish, amphibian, reptile, bird, mammal-primate	Respiratory, flu-like, fever, weight loss
Q-fever	Reptile, bird, mammal	Fever, flu-like
Cryptosporidiosis	Fish, amphibian, reptile, bird	Acute gastrointestinal disturbance, nausea, vomiting, pain, fever, flu-like
Macroparasite infestation, e.g., helminths and ectoparasites	Fish, amphibian, reptile, bird, mammal, mammal-primate	Gastrointestinal disturbance abdominal cramps and pain, weight loss, flu-like
Ringworm	Mammal, mammal-primate	Patchy skin, inflammation, itching
Allergic alveolitis	Bird	Persistent dry cough, chest irritation
Lymphocytic choriomeningitis virus (LCMV)	Mammal	Nausea, vomiting, anorexia, fever, headache, fatigue.
Leishmaniasis	Mammal-dog	Fever, diarrhea, vomiting, respiratory, oral ulceration, cutaneous disease, and secondary bacterial disease.

*If experiencing these indicators report to a healthcare professional. These are a small sample of relatively common animal-to-human diseases. [Table derived from reviewed literature (14)]. Important. The onset of signs and symptoms of an animal-related disease may occur within hours or not for several weeks or months following exposure to an exotic animal. Most cases of diseases are not serious, but it is important to report any suspicion of having an animal-linked disease because treatment may vary from regular illnesses and early access to medical help can alleviate greater problems as well as assist health workers provide best advice*.

#### Disease risk management: animals

The establishment should be registered with a veterinary practice and there should be veterinary input on all aspects of hygiene control (20). Staff should be made aware of zoonotic disease and its transmission (20).

Injured or diseased animals must be assessed and diagnosed by a veterinarian experienced with the species, and any treatment carried out by or under the supervision of such a veterinarian. Diagnoses and guidance on all animal health and disease must be under the control of a veterinarian. A log of all veterinary treatments should be available for inspection (47).

Contact information for all suppliers of animals and foodstuffs, as well as all those to whom animals are handed on (whether or not paid for) must be recorded in order to allow for contact tracing in the event of an epidemiological outbreak (47).

#### Isolation and quarantine facilities

All reasonable precautions must be taken to prevent the outbreak and spread of disease. No animal that is suffering from (or could reasonably be suspected of having come into contact with any other animal suffering from) any infectious disease or which is infested with parasites, shall be brought into or kept on the premises unless effectively isolated and quarantined (20, 53). Species mixing is not advised (20). Consider that diseases can spread two ways and may pass from humans to animals as well as the reverse. A protocol for each species should be available for assessment.

Inspection will assess the length of quarantine given to each new batch of animals. Quarantine periods may vary according to the source and the species. Where animals are acquired from reliable distributors, quarantine may reasonably involve a minimum of 7 days. Where the reliability of supply sources is less well known or unknown, generally longer quarantine periods, for example, 30 days minimum (54, 55) are prudent; the consensus for this review concluded >30 days as a generalized minimum. However, “lower” vertebrate species with, for example, slow disease onset periods (e.g., reptiles) of uncertain origin and health state should be subjected to a minimal of 6 months quarantine (56). Accordingly, avoidance of disease outbreaks may be beneficially related to long quarantine periods, and such longer periods should be applied unless otherwise advised by a veterinarian.

### Food management

Animal foodstuffs should be stored in pest-proof closable/sealable containers to ensure that food does not unduly spoil. Where refrigeration or freezing of food is necessary, facilities should be inspected for both cleanliness and operation (20). Where live-foods are involved, measures should be taken to ensure that containers are as escape-proof as practicable to prevent uncontrolled proliferation and pest issues (20).

Preparation areas must be away from the public and managed so as to avoid cross contamination. Areas used for cleaning, for example, washing of food bowls, should be separate from food preparation areas. Food or drink for human consumption should never be kept in areas intended for storage or preparation of animal feed.

### Safety protocols

#### Supervision

All animals must be under regular supervision (20, 47), which should be as minimally invasive as possible. All animals should be checked no less than twice daily. When premises are closed or otherwise unattended (for example at night in some cases), a supervisor should be available who resides within a reasonable distance from the establishment; independent advice from fire service professionals may assist to determine what is a reasonable distance for a supervisor to be away from a particular establishment in order to provide assistance to fire service personnel if needed. However, preferably, establishments should have onsite supervisors 24/7. The establishment must conform to all safety obligations and maintain contact details for independent 24/7 medical, veterinary and fire safety services that must be clearly available for facility staff.

#### Dangerous wild animals (where applicable)

Where dangerous wild animals are held at an establishment, all enclosures must be both structurally secure and lockable, and animals as well as enclosure locks must be inaccessible to the public. Accordingly, no enclosures should open directly into the public area.

#### Waste disposal

The local authority should be requested to determine suitable waste disposal for the establishment; a list of wastes must be drawn up by the licensee and their disposal agreed with the local authority (20).

A list of wastes may include: soiled animal bedding including feces, urine, and vomit; foods that may have been contaminated; cadavers (dead bodies) of licensed animals, or feed animals; discarded, used animal items such as beds, hides, and toys.

#### Fire safety

Staff must demonstrate to the satisfaction of the fire service or other relevant organization that they are at all times capable of safely evacuating all animals (including animals occupying aquatic enclosures) from the facilities in a manner and time frame as determined by the fire service (47).

A fire risk assessment document should be available for inspection, which will be prepared by the licensee and will list the areas of highest risk, as well as how these risks are mitigated. The fire risk assessment document must include a floor plan and details about fire monitoring equipment, fire fighting equipment, and escape routes. The risk assessment may need to be agreed on by the local fire service if not determined by them. Fire fighting and monitoring equipment must be appropriately serviced and maintained.

#### Electrical safety

All major electrical systems and fixtures should be installed by registered qualified professionals and certified as being safe on a regular basis. Documentary evidence of installation should be available to the satisfaction of inspectors.

New electrical installations or portable equipment must have installation certification available. It is advised that equipment is regularly inspected and is compliant with all relevant local and national regulations.

### License display

Establishments should publicly display a valid operational license, including on their websites (47).

## Animals

An inspection of protocols concerning animal movement, handover and veterinary records is essential along with assessment of the environment and health and welfare of all animals kept at the establishment.

### Register of animals (in/out)

Establishments must maintain a comprehensive and regularly updated register of animals “in” and “out,” together with contact information concerning both the supplier of animals to the establishment as well as recipients to whom animals are handed over by the establishment. This process is intended to assist with the potential requirement for contact-tracing in the event of outbreak of disease (47).

### Veterinary records

Veterinary records must be maintained for all animals that are under veterinary care and treatment (47). New acquirers of animals must be informed of the health history of animals handed over. Records must be maintained for all mortalities. Undiagnosed mortalities should be subject to veterinary pathological examination.

### Transport of animals

All transportation (and handling) of animals under the control of the establishment, including where handed over to acquirers, must be subject to independently approved (for example by a veterinarian) procedures and compliant with relevant legislation.

### Responsible handover

Impulse buying of animals by the public should be discouraged (4). Animals should not be offered for sale as part of special promotions or as part of other product sales (4). The selling of “starter kits” with animals should be discouraged (4). The establishment must ensure that quarantine periods have been met. Inspectors should assess an establishment regarding its general policies toward imparting responsible animal handover and its emphasis on welfare rather than profit. Puppies should be viewed with their mother, and as much verification as possible undertaken to ensure the relationship is genuine (47).

#### Sale of animals/discharge

Animals should be discharged from the premises only when they are in a healthy state. Details of the animal must be provided to the new owner, including the source of the animal, the duration that the animal was held at the establishment, and any historical health or problematic behavioral issues noted and/or treated whilst in the care of the licensee (4). Animals must only be discharged at the species-specific recommended age (47).

#### Information/care guidance

Information provided to new acquirers of animals must be scientific, evidence-based and independently prepared by relevant recognized experts with no conflict of interest in the nature of the business of the establishment (4). The use of “care sheets” should be guarded due to their typical extremely minimalist content, and only utilized where: a. produced by wholly independents experts with no vested interests; and b. clearly stipulated to be considered in association with more substantial overarching independent expert evidence-based guidance (4). Checklists may be helpful to confirm responsibilities for those acquiring animals. Acquirers of animals should be directed to independent impartial biological and veterinary resources for reliable guidance on all aspects of relevant species biology and husbandry (4). Where possible, new owners should provide evidence of adequate husbandry knowledge for the animal that they are acquiring (47).

#### Minimum age of sale/handover

Guiding principles must be that the animal to be handed over meets all legal requirements and is capable of self-feeding, drinking, defecating, urinating, moving independently, and appropriately socialized (47). The animal must be free from disease. Relevant vaccinations must be up-to-date. Very young animals, including amphibians and reptiles, are particularly vulnerable to factors such as dehydration and require the ability to hide in response to noxious stimuli. Where applicable the yolk sac must be fully resorbed.

### Environment

To be especially meaningful and relevant, guidance ought to be structured on a species-specific basis. However, the diversity of species currently available, as well as the variability of their biological needs and an associated dearth of species-specific information, prohibits highly detailed guidance (4). Also, even where reliable information is available, it would be impossible to present this as guidance within the confinement of a single overarching document such as this. From this, the reader can take several key messages:

1. In captive situations, “proper care” is impossible, but responsible management is feasible (57);
2. Species-specific information is essential (58), although typically unavailable, for estimating detailed husbandry;
3. There are no complete biological and behavioral profiles for any species (57);
4. Animals are probably living under conditions of controlled deprivation, even where the best species-specific information is available (59).

Accordingly, although it is impossible to provide useful detailed species-specific information for the diversity of animals involved, this does not negate the potential benefit of providing broad concepts and principles of care that may at least provide some form of standardized recognition of biological needs, welfare and inspection. It is for all these reasons that this guidance exists. However, this guidance is not a “how to care for animals” instructional, nor is it an animal keeping promotional. Animal establishments exist, and these facilities require better-informed structure and inspection. This guidance is intended to assist those with responsibility for such inspections, as well as those managing establishments that are being inspected, by providing an information framework to enable better-informed decision-making and “safety-net” practices to help alleviate especially poor husbandry standards and to promote some fundamental universal management and welfare considerations.

#### Space

All animals ought to be able to express a natural range of behaviors including free movement, locomotion, climbing, play, and burrowing where appropriate for the species (44, 57, 60–65). For some species, sociality is an essential feature of their biology, which may mean that appropriate social groups must be maintained (66). For other species, solitary lifestyles are either usual or advisable due to probable co-occupant aggression (8, 44, 65, 67).

Enclosures must be of a minimum size that is large enough to allow *all* animals to easily use *any* facility (for example, water and feed bowls, heat sources, retreats, basking sites, perches) *at any* one *time*, and allow for all normal behavior and exercise, and adequate escape from conspecifics and the public (8). Aquatic and semi-aquatic species must be able to swim adequately. Arboreal and semi-arboreal species must be able to climb or fly adequately thus the vertical dimension of an enclosure must at least match the horizontal dimension (68). For species that live near water the water depth must allow the animal to dive or drop from a suitably elevated position into water without impacting the floor (69).

##### Calculating minimum space

All animals: floor space must be capable of accommodating a startled animal accelerating, decelerating, and stopping without impacting a boundary (69, 70).

**Figure 1:**
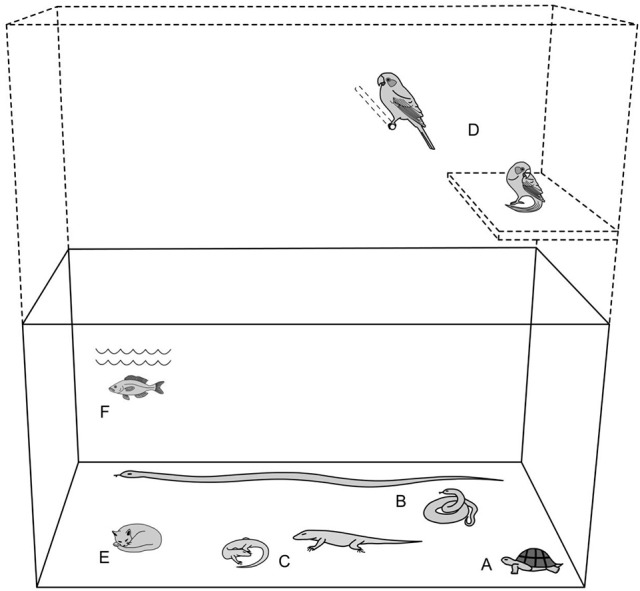
Providing minimum space. [Derived from reviewed literature (8, 20, 32, 57, 61, 65, 68–81)].

Figure 1 provides a methodology for calculating general minimum spatial provisions for enclosure sizes applicable to all animals, utilizing the principle of an animal's “size diameter” X 10 to give a general minimum linear dimension value. The diagram is for dimension purposes only and is intentionally drawn without enrichment or material boundaries.

##### Estimating animal size and applying the system

To estimate the “size diameter” of an animal, either physically measure the animal, or visualize and imagine the animal in a coiled/consolidated or “ball-like” state. For example, a tortoise naturally has an almost “ball-like/consolidated” anatomy and this reveals its “size.” At the other extreme, whereas a snake may be observed in a straight-line posture, by “imagining” the snake in a coiled position, this will provide an approximate finding for its size.

Accordingly, for an animal in this actual or imaginary “ball-like” state, its diameter can be reasonably estimated. When the approximate size-diameter of the animal has been ascertained, the observer may then multiply the diameter by a factor of 10 to calculate the minimum main linear dimension of the enclosure. However, where very small species and individuals of only a few centimeters are involved see “absolute minimum enclosure size.”

The following provides some examples for using the “size-diameter” formula. The diagram is intended to depict examples where all animals featured, despite different forms, have approximately the same “size.” All animals in this example are intended to have a body-size diameter of ~15 cm. The enclosure in the example is intended to illustrate 150 cm primary length and height dimensions, with corresponding depth of 60 cm (= 40% of primary dimensions). Where very small species and individuals are involved see “absolute minimum enclosure size.” The diagram also intentionally does not include furnishings because it is a spatial reference only.

1. Example A. tortoise: shell diameter (naturally approximates a “ball-like” shape) 15 cm; X 10 = 150 cm minimum main linear dimension.
2. Example B. snake: straight-line length 120 cm; coiled (approximates a “ball-like” shape) diameter = 15 cm; X 10 = 150 cm minimum main linear dimension.
3. Example C. lizard: total length 50 cm; coiled (approximates a “ball-like” shape) diameter = 15 cm; X 10 = 150 cm minimum main linear dimension.
4. Example D. bird: total length 20 cm; “rolled-up” (approximates a “ball-like” shape) diameter = 15 cm; X 10 = 150 cm minimum main linear dimension.
5. Example E. kitten: total length 35 cm; “rolled-up” (approximates a “ball-like” shape) diameter = 15 cm; X 10 = 150 cm minimum main linear dimension.
6. Example F. fish: total length 30 cm diameter (naturally approximates a “ball-like” shape) = 15 cm; X 10 = 150 cm minimum main linear dimension.
7. Note: the tortoise (A), the kitten (E), and the fish (F) images provide examples of animals that are already “ball-like” in image/anatomy, thus these are represented using a single illustration each.

Figure 1 represents minimum spatial accommodation. This system ensures at least one “safe” minimum primary dimensions (e.g., main linear and vertical in example). Common sense and reasonable practice determine that although one dimension may be calculated to attain standard minimum spatial provision, this should not be taken to suggest that enclosures maybe elongated and extremely narrow. Reasonable proportionality should be applied using the calculated minimum length or height as a guide. For arboreal and flying species, which may regularly use both ground and elevated positions, the enclosure should comply with the same calculation formula for both vertical and horizontal dimensions. Birds must be able to fly without their wings contacting the sides of the enclosure.

##### Absolute minimum enclosure size

Where very small species and individuals are involved one must apply the principle of “absolute minimum” enclosure size to prevent, for example, animals measuring only a few centimeters being confined to “micro-environments,” which should be discouraged.

Save as for short-term transportation, no enclosure should be <100 cm at its main linear dimension (82–85), with other dimensions measuring no <40% of this figure, and where arboreal species are concerned, the vertical dimension should also measure no <100 cm proportionately.

#### Stocking densities

The spatial minimum system above (Figure 1) should be adopted in association with the guidance on “Overcrowding and crypto-overcrowding” to assess permissible maximum stocking densities (8).

##### Overcrowding and crypto-overcrowding

Overcrowding can be considered as having two “forms” “overt overcrowding” and “covert (or “crypto”) overcrowding” (8). The number of animals occupying a certain amount of space may allow overt overcrowding to be estimated (8). Whereas crypto-overcrowding essentially refers to the availability of all facilities to all animals when they require access to those facilities (8). Accordingly, an enclosure that appears large and abundant but that lacks the ability to “service” all animals' needs at any time is capable of being overcrowded by its inherent deficiency (8, 69). Therefore, in order that a space is not overcrowded it must allow both space to roam as well as possess facilities, e.g., a water bowl or basking site that all animals together at any time can simultaneously use (8).

Note: Summary principles for spatial issues and enclosure size.

Estimate animal's body-size diameter in centimeters; multiply diameter by 10 to determine minimum required primary enclosure dimension (e.g., length or height). All other dimensions to be reasonably proportionate to primary dimension (e.g., no less that 40% of primary dimension). No enclosure to have primary dimension <100 cm.

Stocking density: Apply “crypto-overcrowding” principle [i.e., all animals must be able to use all facilities/furnishings (e.g., water bowls, bathing pools, perches, hides, basking sites) at any one time]. Where an enclosure meets this principle its stocking density has not been breached.

#### Temperatures, heating, lighting and humidity

In the following sections we have adopted deliberately broad criteria utilizing objective data pertaining to heat, light and humidity observed in the natural environment on a “zone-by-zone” basis—an approach used as broad guidance in reviewed literature [for example (44, 57, 58)]. In effect, we have considered the approximate possible ranges of heat, light and humidity to which animals may be exposed depending on region of the world and their essential habits. This approach is extremely challenging because of the inevitable diversity of heat and light levels even within a relatively specific environment, and because the needs of animals also vary substantially, not only on a species-specific basis, but also depending on season, animal condition and concomitant biological need.

The levels of, for example, heat and ultraviolet light, in nature may be extreme and avoided by animals at certain times. At other times, suboptimal levels of heat and light may result in deficiencies that cause animals to adopt compensation strategies when environmental resources are more abundant (86). Few detailed guidance reports are available pertaining to recommended levels of UV, although there is some highly limited information for reptiles and amphibians [for example (44, 58, 87, 88)]. In this document we have adopted an ultraviolet light index (UVI) of 0 (zero UV) to 13 (high).

Accordingly, considerable caution must be applied when using thermal and UVI maximums, because these are unlikely to represent species optimal levels (58). Therefore, we have used lower-to middle natural range thermal and UVI indicators to suggest possible “safety net” guidance. Nevertheless, it should be emphasized that the rationale for, in particular, consideration of thermal and UV guidance in this document is that the parameters should be *accessible*, but *not imposed* conditions, causing animals to have the ability to voluntarily choose both thermal and UV gradation as much as possible.

Lighting must be appropriate to the species and this should include both appropriate periodicity of lighting as well as take account of diurnal and nocturnal habits (20, 58, 89). All animals require adequate levels of restful sleep for optimum health (67, 90). Accordingly, diurnal species that would normally sleep at night should not be exposed to invasive lighting during this time. Light and heat should not be mutually dependent so that necessary heat is lost when lights are deactivated (20). While hiding areas are important for seclusion, “light-management” should not be relegated only to an animal's ability to withdraw from light. It is always preferable that the ambient environment (in terms of lighting) should be adequately controlled, simulating, as much as possible, natural cycles of light, and dark (58).

For many species, light (in its various forms) is an important component of health (91, 92). Light, its type, intensity and periodicity, influences physiological, behavioral and psychological states, as well as aids in the control of topical infections. Too little light of the correct type can result in, among other things, stress, decreased activity and metabolism, and disease. Furthermore, too much light of any form can result in environments that are “photo-invasive” and stressful, and can cause a range of health problems (58, 67, 92). Ultraviolet (UV) light has varying degrees of significance according to species, and for some species prolonged exposure to UV can be harmful (58). Typically, broad-spectrum “UVB” bulbs are used in artificial environments, and there are many forms available (58).

“Open habitat” (such as grassland/prairie and desert) species are naturally exposed to high intensity UV light. These raised levels of UV light are frequently important to these species, and typically these animals possess physiological mechanisms to regulate their condition under such exposure.

“Closed habitat” (e.g., forest, subterranean, and aquatic) species are naturally exposed to lower intensity UV light, and intensive UV exposure may result in eye injuries and otherwise compromise health. High-level exposure to cage-based UV can also harm the human observer's eyes.

Essential UV light requirements may not correspond with the number of daylight hours. In nature, some animals, while active during the day, may not be directly exposed to strong or significant sunlight during this entire period whether due to habitat cover or deliberate heat or light avoidance (for example, reptiles shuttling between sunlight and shade during normal patterns of thermoregulation). Relatedly, captive animal management (according to species) may involve deliberate UV exposure for only a portion of any single day.

Diurnal species requiring UVB lighting should have appropriate UVB emitting lamps. These should be replaced according to manufacturer's recommendations. Mercury vapor or metal halide UVB emitting lamps may also be used to provide a daytime heat source. UV light sources must not be screened by non-UV transmitting glass or plastic (because glass or plastic will absorb or negate virtually all UVB light). The distance from the UV radiation source must not be so close as to expose animals to excessive radiation, nor should it be for excessive periods. Animals should have areas of shade so that they can escape from the light if desired. No species should be subject to persistent invasive light (light bulbs should not be exclusive heat sources and interfere with normal rest). A day/night time period of (50% light/50 dark) is a broad guide, although crepuscular and nocturnal species' habits should be carefully considered.

It is vital that lighting needs according to species are carefully managed. For example, nocturnal, crepuscular (twilight) and subterranean species should not be exposed to excessive levels or periods of light. Nocturnal species in particular, should not be exposed to invasive light on the basis of any display needs of the establishment. While it is important to position enclosures for light-sensitive species in less-invasive areas, this should not be taken to imply merely positioning cages in “dark” areas of a facility. Nocturnal and crepuscular species should have limited light sources dedicated to primarily low-level lighting that includes some UV component. Brighter areas and “warm” basking spots are acceptable, as long as these facilities are not major components of the environment. Significant specific welfare considerations arise in relation to nocturnal and crepuscular species because human activities frequently involve risk of disturbance and stress to animals, and also because keepers are commonly resting during animals' activity period this means that reasonable opportunities for observing normal health and welfare may be confounded.

Optimum temperatures, normal ranges and critical thermal maximums vary considerably with species. Also, constant uniform temperatures (i.e., a single “set” temperature of, for instance 25°C, across the entire enclosure) are not consistent with good practice in most cases and may result in stress and harm to animals. Therefore, a reasonable variation of temperature within each enclosure may be important for animal health and welfare. Accordingly, these guidelines adopt a precautionary approach. Inspector and establishment manager familiarity with species natural history is an important component of assessment and management.

Humidity is a feature of air-quality and can be important to animal health and welfare (58, 67, 90). Characteristically, desert-dwelling species are adapted to drier low-humidity environments whereas aquatic and rainforest species are adapted to wetter high-humidity environments. Inappropriate humidity can result in stress and disease (58, 67, 90). Table 2 provides generalized climate zones (daytime air temperatures) as a rough guide to basic humidity values.

**Table 2:** Default (“safety net”) guide to temperature and humidity provisions for terrestrial and aquatic animals.

**Zone/climate**	**Temperatureday**	**Temperaturenight**	**Humidity**
Temperate	15–20°C	12–18°C	60–70%
Subtropical	20–25°C	18–20°C	60–70%
Tropical	25–30°C	24–27°C	70–80%
Semi-arid/desert	20–25°C	18–20°C	40–60%
Arid/desert	25–30°+C	21–24°C	30–45%
Dogs/cats	10–26°C	10–26°C	30–45%
Freshwater (cold)	10–15°C	10–15°C	N/A^*^
Freshwater (temperate)	10–25°C	10–25°C	N/A^*^
Freshwater (subtropical)	16–22°C	16–22°C	N/A^*^
Freshwater (tropical)	24–27°C	24–27°C	N/A^*^
Marine (cold)	7–12°C	7–12°C	N/A^*^
Marine (temperate)	10–18°C	10–18°C	N/A^*^
Marine (subtropical)	18–22°C	18–22°C	N/A^*^
Marine (tropical)	22–28°C	22–28°C	N/A^*^

Derived from reviewed literature (8, 44, 57, 58, 93–99). Table should not be used to determine regular thermal management practices. The main purpose of the table is to offer “safety-net” temperatures.

* *Humidity levels are not relevant under water*.

##### Basking sites and heat sources

It is important that basking animals are able to warm their bodies across their entire length as would occur from sunlight in nature, whether in relation to warmth or ultraviolet light acquisition as appropriate (8). For example, several heat sources may be required to span the length of a large lizard or snake; thus should a heat lamp provide only a “spot” on one section of that animal then the heat source should be considered inadequate.

Poorly conceived or instituted conditions relating to temperatures, ultraviolet light, basking options, humidity, and ventilation can be harmful or lethal to animals (8, 58). Similarly, poorly situated provisions (e.g., heat sources) can also result in harm or death (8). Furthermore, many animals, notably ectothermic species such as amphibians and reptiles, require subtle thermal gradients rather than single “constant” temperature environments; and a single “hot spot” in an otherwise thermally bland area in a cage may not offer sufficient variation (8, 58).

Temperatures, UV levels, and humidity should be recorded in the establishment and assessed against acceptable ranges. The housing of any relevant species outside the given range of temperatures should “red-flag” conditions as imminently potentially dangerous. The principle for assessing a minimum standard adequate thermal range should be determined by evidence-based species thermal preferences and/or natural geographic and thermal ranges of species derived from relevant climate map data for regions. Enclosures should possess a thermal gradient (warmer and cooler areas) appropriate to the species.

##### Light and lighting periodicity

Care should be taken regarding both the period of exposure and the placement of light sources to avoid over-exposure and thermal burns (8, 44). For most species, both a daytime (photophase) and a night-time (scotophase) period are important within each 24-h cycle (8). Similar to the good practice of providing a thermal gradient (range of available temperatures within a single enclosure), light (general environmental ultraviolet where appropriate) should also offer a range of intensities rather than a uniform photo-environment (44, 58). Table 3 offers a default (“safety net”) guide to lighting and its periodicity for animals.

**Table 3:** Default (“safety net”) guide to lighting periodicity.

**Natural habitat type**	**Natural lifestyle type**	**Periodicity**	
		**Daytime (h)**	**Nighttime (h)**
**Open habitat**	Diurnal	12–16	8–12
	Crepuscular (phased transition)	4	4
	Nocturnal	12	12
**Closed habitat**	Diurnal	12	12
	Crepuscular (phased transition)	4	4
	Nocturnal	12	12

*Derived from reviewed literature (8, 44, 57, 58, 93–99)*.

Electric UV bulbs should be checked regularly for their output because the level of UV output will decrease over time. Furthermore, all lighting equipment must be kept free of dust; this is particularly important for UV lighting where accumulated dust will act to absorb the UV and convert it to heat. Hand-held UV-monitoring devices are available to establish bulb and environment UV intensity. Table 4 offers a default (“safety net”) guide to UV and its intensity for animals based on data derived from primary natural solar radiation in different climates; subterranean and aquatic environments therefore may receive diffuse, little, or no exposure.

**Table 4:** Default (“safety net”) guide to ultraviolet light (UV) exposure for species from different climates/habitat types.

**Zone/climate**	**Habitat type**	**Intensity in nature values**	**Captive (“safety net”) values**		
			**Diur**	**Crep**	**Noct**
*Equatorial*	Tropical forest (rainforest)	3.5–13	2.5–5	0.5–1	0–3
	Subtropical/tropical swamp forest	3–9	2–5	0.5–1	0–3
	Subtropical/tropical dry forest	3–9	2–5	0.5–1	0–3
*Arid*	Arid desert	3.5–13	2.5–5	0.5–1	0–3
	Semi-arid desert	3–9	2–5	0.5–1	0–3
*Semi-arid/Mediterranean*	Dry savanna	3–11	2–5	0.5–1	0–3
	Savanna	3–11	2–5	0.5–1	0–3
*Marine*	Tropical marine	3–7	N/A	N/A	N/A
	Coral Reef	4.5–13	3.5–5	0.5–1	0–3
*Temperate*	Temperate forest & woodland	3–5	2–4	0.5–1	0–3
	Temperate marine	3–6	N/A	N/A	N/A

*Derived from reviewed literature (8, 44, 57, 58, 93–99). Diur, Diurnal; Crep, Crepuscular; Noct, Nocturnal; Ultraviolet light is potentially harmful at both low and high levels. However, whereas UV deficiency may be chronically harmful, UV excess may be both acutely and chronically harmful. The “Intensity in nature values” used in Table represent objective UV levels across relevant world zones recorded at midday including seasonal variation (100). Therefore, the values given represent high UV levels, which many animals would avoid. The “Captive (safety net) values” are derived from lower seasonal midday values minus one index point as a safety margin. The upper value is intended to indicate the maximum UV intensity (i.e., a thermal-basking/UV) “hotspot” within an environment and not the general ambient UV conditions. UV should be measured at both elevated basking sites and at ground level. Accordingly, the output of an artificial UV source (which may decline over time) must be calculated to correspond with the safe values in Table 4, and imply levels at a focal heat and UV site, and does not imply utilizing these levels in the general environment*.

Daytime-to-night-time transitions (and vice-versa) that are gradual (such as a progressed by dimmer/control switching) are preferable to sudden “on-off” transitions.

#### Sound and noise

Sounds or noises may be gross or subtle elements in an environment that should be carefully assessed for their invasive potential (101). Road traffic, machinery, “white goods” (e.g., refrigerators) and voices may all be relevant audio polluters and stressors. Many, if not most, animals are sensitive and often stressed by invasive sound and noise (102, 103, 104). Relatedly, because different species may be sensitive to different (e.g., airborne, terrestrial, and seismic vibration or aquatic) sounds, an awareness of both species audio sensitivities and environmental noise pollution is essential.

Given the diversity of species in trade and keeping along with respective varying audio sensitivities (as well as information deficits), it is not possible to provide detailed guidance on acceptable sound or “noise” levels. However, broadly speaking, Table 5 may offer general guidance and comparative context regarding audio sensitivities for various animals. Accordingly, Table 5 indicates what certain animals probably can hear or sense, and therefore, what frequencies and intensities of sound may be or probably are invasive. Essentially, sounds audible to species kept (especially higher decibel sounds) should be prevented, and the general environment should be considered as a “quiet place”.

**Table 5:** Default guide to sound and noise perception in animals.

**Animal**	**Audible range frequency = Hertz(Hz) kilohertz (kHz)**	**Intensity level = Decibels**
Invertebrates		
Arthropods	up to 150 Hz	0–10
Marine	500 Hz−2 kHz	0–100
Fishes	1–3 kHz	0–40+
Amphibians	200 Hz−3 kHz	10–60
Reptiles		
Snakes	100–700 Hz	40–50
Lizards	1–3 kHz	40–50
Turtles	200 Hz−1.2 kHz	40–50
Birds	1–4 kHz	0–10 (occasionally 100)
Mammals		
Rabbits	360 Hz−42 kHz	20
Guinea pigs	50 kHz	20
Small prey mammals	20 Hz−85 kHz	20
Dogs	67 Hz−45 kHz	0–95
Cats	45 Hz−91 kHz	20
Humans (for comparative purposes)	20 Hz−20 kHz	60

*Derived from reviewed literature (28, 65, 104–112)*.

#### Ventilation

Ventilation involves the process (or management) of the medium of air as it affects an animal (20, 113). Ventilation in the establishment environment and facilities requires addressing both the biological needs of animals as well as the practical aspects of cage positioning in the wider environment, and may require considerable planning (20). Movement of gasses, airborne particles and heat extraction are also features of ventilation. A variety of mechanical ventilators are available to assist with specific air quality provision.

In essence, air can be thought of as a mixture of gasses and a very light “fluid” environment—almost as if living in very “thin” water. Just as water quality is important to organisms that occupy the aquatic environment, so too air quality is important to animals that occupy the terrestrial and arboreal environments. While many animals, such as domesticated species comfortably share air qualities that match human preferences, others such as some amphibians and reptiles may require highly specific air qualities that differ from typical human preferences. For example, humid, very warm and “static” air environments may be stressful to species adapted to occupying elevated “breezy” arboreal sites. In addition, temperature change, excitement, activity, and processes such as skin-shedding can produce marked respiratory changes in animals that must be accommodated by adequate environmental ventilation. Knowledge of species-specific natural history is important to providing adequate ventilation and to its assessment.

The starting point for adequate ventilation is the general environment of the establishment itself. Unless enclosures are externally ventilated (which is unlikely) then the establishment's ambient air must be of both sufficient quality and throughput. As indicated under “Hygiene,” the presence of odors (especially if malodorous) usually is an indicator of poor hygiene and poor facility ventilation. Further, enclosures or other items should not be in positions as to negatively compromise the airflow. As a very basic guide, species with possibly greater thermal and humidity sensitivities, while still being well ventilated, should be maintained in areas that are especially well protected from door drafts, and no species should be subject to “cold air bursts”. Fewer enclosures promote better general ventilation. No smoking of tobacco should occur within the vicinity of indoor animals on the precautionary basis that “passive” inhalation may impact on animal health.

Little species-specific information is available concerning recommended ventilation levels for the wide variety of animals kept in captivity. However, information regarding laboratory animals (rodents and lagomorphs) held in relatively large numbers in open cages (with consequential relatively high oxygen demands) indicates that general room ventilation should involve 15–20 air exchanges per hour (28). Because this air exchange rate applies to species and numbers of animals that require considerable ventilation, it is likely that this rate is also suitable for other species under normal densities including groups of enclosures, provided that all enclosures have adequate air access ways.

### Environmental enrichment

Furnishings must reflect the habitat and behavioral needs of animals, for example in respect of: terrestrial, burrowing, subterranean, arboreal, climbing, postural-positional, semi-aquatic, aquatic characteristics (114). Accordingly, plant life (real or simulated), terrestrial mounds, deep substrates, rocks, as appropriate for the species should be provided in all environments (81, 115). Bare and under-stimulating environments should not be utilized (44, 116, 117). In addition, many animals (including, for example, reptiles, and birds) are known to play, and may require safe “toy” objects to occupy them (118).

#### Exercise facilities

The issue of space and spatial needs are addressed more specifically elsewhere in this document. However, although spatial issues and exercise are clearly conjoined subjects in certain respects, in some circumstances they may be more distinct. Therefore, exercise facilities will be dependent on the species held and may or may not be sufficient as part of the enclosure.

Many wild animal species naturally occupy large home ranges (44) that may be considerably greater than those of domesticated animals (8, 44). Also, many small species and juveniles or small individuals of large species may be as active as adults and sometimes more so (8, 67). Some species, for example bearded dragons, may or may not seek to wander significant distances on a given day, so their “exercise” needs may be unclear, although additional roaming space beyond that provided by the vivarium may be important. For other animals, for example, dogs, exercise arrangements must be in place, which may involve formal lead walks or time in allocated play areas.

Certain species, for example hamsters, in the closed environment may arguably be considered to exercise reasonably using running wheels. Fish are able to exercise in their aquarium, but consideration for size of aquarium will be dictated by the needs of the species. Obviously sedentary and reclusive species such as coral fish may require less space than most freshwater river species, but this does not imply that restrictive minimalist environments are justified.

Details of frequency and time of exercise must be recorded. Inspection of exercise areas will be included and evidence of staff exercise rosters and individual exercise logs must be kept.

#### Substrates/bedding

Substrates or “beddings” are important or vital for many species as a medium for appropriate behavior, such as hiding and burrowing, as well as for the avoidance of injury when “diving” to the ground from elevated positions, and also for the absorption of waste (117, 119). Under natural conditions, biotic (such as microbes) and abiotic (such as climatic) factors control substrate quality. In artificial environments, this natural “cleaning” process is greatly compromised, making human management of substrate very important (117). While it may be tempting to the animal manager to opt for what are often perceived as “easy” substrates (such as newspaper), these materials provide poorly for behavioral needs and indicate lazy management. Furthermore, because these materials are often sourced from public donations, there is no guarantee that they are free from relevant contaminants and potentially toxic compounds. Accordingly, well-managed naturalistic substrates are preferable to clinical conditions other than in exceptional situations, such as specific laboratory or veterinary protocols and are often essential for animal welfare (117). Table 6 offers “default” guidance on substrates for animals (44, 117).

**Table 6:** Default substrates/bedding for animals.

**Species habitat type**	**Enclosure substrate**
Open habitat species	Soft (play-pit) sand with uncontaminated (e.g., pesticide-free etc.) topsoil @ 50/50 ratio; hay; sphagnum moss; pure/plain (e.g., pesticide-, fertilizer- and vermiculite-free) soil/peat moss.
Closed habitat species	Aspen and pine shavings; sphagnum moss; pure/plain (e.g., pesticide-, fertilizer- and vermiculite-free) soil/peat moss.
Subterranean/burrowing species	Soft (play-pit) sand mixed with sphagnum moss; pure/plain (e.g., pesticide-, fertilizer- and vermiculite-free) soil/peat moss; dried Spanish moss in small quantities.
Arboreal species	Aspen and pine shavings; sphagnum moss; pure/plain (e.g., pesticide-, fertilizer- and vermiculite-free) soil/peat moss.
Aquatic & semi-aquatic species	Fully aquatic species require, as environmental enrichment, a base substrate such as gravel or sand, although in very large enclosures and pools, microbially balanced detritus may be appropriate or beneficial. For certain bottom feeding species substrates may also be essential to provide an acceptably naturalistic environment for dietary habits. Substrate-free aquaria may be acceptable only when used for species that do not behaviorally interact with such media for any purposes. Semi-aquatic species should have submerged and dry areas of sand, gravel (too large to ingest) or stone (or combinations) to enable interaction for purposes of display, feeding or seclusion.
Domesticated dogs & cats	Absorbent floor coverings may include newspaper, shavings or hay/straw, but must be changed regularly to avoid soiling and wetting. Soft beddings such as proprietary items, blankets, towels, specialized materials must be available and regularly laundered.

*Derived from reviewed literature (8, 44, 57, 58). Substrates should be completely removed and discarded several times monthly. The enclosure must be cleaned prior to introducing fresh substrate*.

Gravel, hemp, wood chips, along with numerous commercial product substrates present highly problematic potentially ingestible materials that should not be used. In addition, certain materials, e.g., cedar wood and certain barks can be toxic. Although some of these do occur under natural conditions for many species, captive animals often ingest harmful substrate incidentally or develop eating disorders termed “pica” and may consume small stones and other items resulting in illness and death.

#### Sleeping/hide areas

Sleep and rest are fundamental biological needs for all animals and an essential requirement to health (57, 90). Good “quality” rest requires seclusion, and thus the provision of appropriate hiding places, which may also include appropriate substrate, as well as freedom from invasive disturbances including light, noise, co-occupant activity, extraneous movement, handling, cage-pests, hunger, thirst, and other factors (57, 91, 92).

##### Transparent boundaries

Transparent boundaries (such as glass) commonly result in psychological stress and behavioral and physical problems for certain animals, for example, reptiles (57, 69). Where enclosures involve transparent sides these should be masked wherever possible so that the boundary appears “real” and visible to the animal.

### Nutrition—food and water

#### Food and water hygiene

Written protocols for food and water provision for each species must be available (57, 58). This must include types of feed, method of water provision, frequencies, preparation, and cleaning.

Food must be prepared in a clean and hygienic area dedicated for that purpose. Hot and cold water must be available. Contamination to and from other cleaning activities and other food preparation areas (for example staff facilities) must be avoided. Water must be fresh. Where tap water is used it should preferably be filtered or have been left to stand for 24 h to reduce possible chlorine products. Food and water vessels must be cleansed with appropriate cleaning agents at least several times weekly.

#### Food and water availability and appropriateness

Food and water should always be clean and obviously fresh. Considerable diversity exists for feeding habits, and diet must be suitable for the species and provided at sufficient frequency (20, 58). Although some animals rarely or possibly never drink standing water (20), it should always available for every animal even where this may involve a small, accessible, water bowl for arboreal or extreme desert-dwelling species. Atomised water may be sprayed into an enclosure during the mornings several times per week to reflect dew, which certain species imbibe (20). For some species, this same process may be used to “mist” the bodies of animals, which then imbibe droplets (20). Shallow pools should be provided for bathing and non-bathing species alike, and body-misting of many species (including reptiles and birds) may be important several times per week.

Water quality for aquatic and semi-aquatic species is important and requires regular monitoring, and a regular water testing protocol should be in place to test for basic parameters of pH, nitrite, nitrate, free-ammonium, and dissolved oxygen (20). Where appropriate hardness and salinity testing should be added.

Appropriate food and water provisions should be made available for all live invertebrates intended as food (20).

### Welfare assessment

Establishment staff should remain constantly and proactively alert for early onset of problems, including injury, ill-health, behavioral, and general environmental problems. The number of animals in an establishment should be proportionate to the ability of the staff to identify emerging problems. Animal welfare may be assessed in different ways, including: behavioral signs (i.e., what animals “do”); physical signs (e.g., injury); physiological signs (e.g., blood, organ tissue; feces, and urine tests); and physical examination (e.g., handling and clinical assessment). Over 13,000 animal species (4) are kept in captivity, and there is a lack of species-specific baseline physiological data for the great majority of this diversity. Physiological measurements are also often indeterminate for welfare among captive animals, for example due to the similarities between certain stress states [e.g., captivity-stress and excitement for food (8, 120)]. Also, invasive physiological investigations and physical handling may involve stress, as well as lack practical feasibility in most situations. Behavior is widely accepted to provide a reliable method for welfare assessment, and both behavior and physical signs allow for overt observation and minimal invasiveness (8, 57, 69, 120). Accordingly, in this article we have focused on behavioral and certain overt physical signs to assess animal welfare.

Tables 7A–F provide basic guidance on using behavioral signs for the identification of stress, and Table 8 provides basic guidance identifying physical signs of discomfort, as well as and manifestations of injury and disease.

**Table 7A:** Example welfare/behavioral criteria for assessment of stress: some key signs: invertebrates.

**Behavioral sign**	**Cause/Problem**
Lethargy	1, 6
Hyperactivity	1, 5
Release of urticating hairs (some tarantulas)	1, 3
Aggression	1, 3
Anorexia/reduced response to food/refusal to feed	1, 3

*Derived from reviewed literature (1, 8, 44, 57, 79, 81, 115, 116)*.

Cause/problem keys:

^1^*Overly restrictive/incorrect environment/inability to hide/retreat. ^2^Co-occupant aggression/harassment. ^3^Fear/defense. ^4^Pain/trauma/disease. ^5^Environmental stressor e.g., hyperthermia. ^6^Environmental stressor e.g., hypothermia. ^7^Environmental stressor e.g., hypoxia. ^8^Hunger. ^9^Environmental stressor e.g., incorrect humidity. ^10^Excessive handling. ^11^Learned helplessness' (includes apparently normal behaviors in highly adverse conditions). ^12^Under-stimulation*.

**Table 7B:** Example welfare/behavioral criteria for assessment of stress: some key signs: fishes.

**Behavioral sign**	**Cause/Problem**
Congregating at surface	1, 4, 7, 8
“Gasping” at surface	1, 4, 7
Rapid opercular (“gill-covers”) movement	1, 4, 7
Avoidance behavior, hiding from light, others	1, 2, 3, 4
“Flashing” (darting moves)	1, 4
Rubbing against objects	1, 4
Anorexia/reduced response to food	1, 4

*Derived from reviewed literature (1, 8, 44, 57, 79, 81, 115, 116)*.

Cause/problem keys:

^1^*Overly restrictive/incorrect environment/inability to hide/retreat. ^2^Co-occupant aggression/harassment. ^3^Fear/defense. ^4^Pain/trauma/disease. ^5^Environmental stressor e.g., hyperthermia. ^6^Environmental stressor e.g., hypothermia. ^7^Environmental stressor e.g., hypoxia. ^8^Hunger. ^9^Environmental stressor e.g., incorrect humidity. ^10^Excessive handling. ^11^Learned helplessness' (includes apparently normal behaviors in highly adverse conditions). ^12^Under-stimulation*.

**Table 7C:** Example welfare/behavioral criteria for assessment of stress: some key signs: amphibians.

**Behavioral sign**	**Cause/Problem**
Rapid body movements, such as jumping and climbing with falling	1, 2
Body “flattened” against cage floor, lethargy	1, 2, 4, 5, 6, 9
Closed eyes	1, 4, 5, 6, 9
Lethargy/reduced responsiveness	1, 4, 5, 6

*Derived from reviewed literature (1, 8, 44, 57, 79, 81, 115, 116)*.

Cause/problem keys:

^1^*Overly restrictive/incorrect environment/inability to hide/retreat. ^2^Co-occupant aggression/harassment. ^3^Fear/defense. ^4^Pain/trauma/disease. ^5^Environmental stressor e.g., hyperthermia. ^6^Environmental stressor e.g., hypothermia. ^7^Environmental stressor e.g., hypoxia. ^8^Hunger. ^9^Environmental stressor e.g., incorrect humidity. ^10^Excessive handling. ^11^Learned helplessness” (includes apparently normal behaviors in highly adverse conditions). ^12^Under-stimulation*.

**Table 7D:** Example welfare/behavioral criteria for assessment of stress: some key signs: reptiles.

**Behavioral sign**	**Cause/Problem**
Interaction with transparent boundaries (ITB): frequent interaction/attempts e.g., scratch, “pace,” “climb,” rub against cage-glass	1, 12
Hyperactivity: moderate/greater locomotor activity/escape attempts e.g., “pacing” perimeter, digging, climbing	1, 2, 5, 12
Hypoactivity/sedentary behavior	1, 2, 3, 4, 6, 12
Avoidance behavior: e.g., fleeing from co-occupants, head-hiding	1, 2, 3
Hissing	1, 2, 3
Inflation of the body	1, 2, 3
Repeated inflation and deflation of the body	1, 2, 3
Repeated inflation and deflation of the throat	1, 2, 3
Open-mouth breathing (rapid or slow)	1, 2, 3, 4, 5
Voluntary regurgitation of food	1, 2, 3, 4, 6
Rapid pigmentation change	1, 2, 3, 5
Biting/cannibalism	1, 9, 12

*Derived from reviewed literature (1, 8, 44, 57, 79, 81, 115, 116)*.

Cause/problem keys:

^1^*Overly restrictive/incorrect environment/inability to hide/retreat. ^2^Co-occupant aggression/harassment. ^3^Fear/defense. ^4^Pain/trauma/disease. ^5^Environmental stressor e.g., hyperthermia. ^6^Environmental stressor e.g., hypothermia. ^7^Environmental stressor e.g., hypoxia. ^8^Hunger. ^9^Environmental stressor e.g., incorrect humidity. ^10^Excessive handling. ^11^Learned helplessness” (includes apparently normal behaviors in highly adverse conditions). ^12^Under-stimulation*.

**Table 7E:** Example welfare/behavioral criteria for assessment of stress: some key signs: birds.

**Behavioral sign**	**Cause/Problem**
Pacing; route-tracing of cage	1, 2, 11, 12
Head bobbing, spot-pecking (at point on others or items but not to eat)	1, 12
Huddled with consistently ruffled feathers and drooping wings	4
Self-plucking (with damaged/missing feathers, bald areas, skin lesions)	1, 12
Unable to stand	2, 4
Lunges and/or flies at cage bars repeatedly	2, 3, 4
A “perching” species not using perches	1, 3, 4
Fighting	1, 2
Vocalization/emits distress calls repeatedly	2, 3
Blood on bird and/or perches	1, 2, 3, 4
Lethargy	1, 2, 3, 4, 11, 12
Cowering, attempting to hide, attempts to dig/climb/escape from cage	2, 3, 4, 10, 11

*Derived from reviewed literature (1, 8, 44, 57, 79, 81, 115, 116)*.

Cause/problem keys:

^1^*Overly restrictive/incorrect environment/inability to hide/retreat. ^2^Co-occupant aggression/harassment. ^3^Fear/defense. ^4^Pain/trauma/disease. ^5^Environmental stressor e.g., hyperthermia. ^6^Environmental stressor e.g., hypothermia. ^7^Environmental stressor e.g., hypoxia. ^8^Hunger. ^9^Environmental stressor e.g., incorrect humidity. ^10^Excessive handling. ^11^Learned helplessness” (includes apparently normal behaviors in highly adverse conditions). ^12^Under-stimulation*.

**Table 7F:** Example welfare/behavioral criteria for assessment of stress: some key signs: mammals.

**Behavioral sign**	**Cause/Problem**
Compulsive i.e., repetitive, apparently functionless behaviors: e.g., pacing, figure of eights, circling, spinning, self-mutilation, over grooming	1, 2, 3, 4, 10, 11, 12
Withdrawal, reduced responses, lethargy, vocalization	1, 2, 3, 4, 10, 11, 12
Aggression to humans or conspecifics	1, 2, 3, 4, 8, 10
Cowering, attempting to hide, attempts to dig/climb/escape from cage	1, 2, 3, 4, 8, 10

*Derived from reviewed literature (1, 8, 44, 57, 79, 81, 115, 116)*.

Cause/problem keys:

^1^*Overly restrictive/incorrect environment/inability to hide/retreat. ^2^Co-occupant aggression/harassment. ^3^Fear/defense. ^4^Pain/trauma/disease. ^5^Environmental stressor e.g., hyperthermia. ^6^Environmental stressor e.g., hypothermia. ^7^Environmental stressor e.g., hypoxia. ^8^Hunger. ^9^Environmental stressor e.g., incorrect humidity. ^10^Excessive handling. ^11^Learned helplessness” (includes apparently normal behaviors in highly adverse conditions). ^12^Under-stimulation*.

**Table 8:** Example welfare/physical signs of injury or ill health and possible causes.

**Signs**	**Problem**	**Cause**
Open mouth breathing	Hyperthermia; disease; major head/neck injury	Critically high temperature; infection/organic dysfunction; fall; drop; co-occupant attack; transport trauma
Panting	Hyperthermia	Too high temperature
Sores on head, neck, or dorsal region	Thermal burns	Too close or too hot “hot-spot” (basking lamp) often combined with too low environmental temperate
Hyperactivity	Hyperthermia	Too high temperature
Hypoactivity; anorexia	Hypothermia; disease; injury; pain	Too low temperature; infection/organic dysfunction; fall; drop; co-occupant attack; transport trauma
Emaciated appearance	Starvation/dehydration; chronic injury/disease	Infection/organic dysfunction; fall; drop; co-occupant attack; transport trauma
Uncharacteristic red or white patches on head, skin, extremities	Injury; disease	Infection/organic dysfunction; fall; drop; co-occupant attack. Attack by prey insects e.g., crickets
Deformities	Malnutrition, Injury	Metabolic Bone Disease. Co-occupant aggression. Trauma
Incomplete skin shed	Poor humidity. Poor environmental enrichment	To low humidity. Lack of provision of shedding aids
Damage to extremities. Especially tail tip and toes	Poor shedding, injury, trauma	Co-occupant aggression. Poor handling may induce tail drop in some lizard species. Incomplete shedding can damage toes in some lizard species.
Injuries anywhere on body	Attacks by co-occupants or self-harm	Co-occupant aggression, invasive courtship routines, hunger, inability to avoid cage-mates when required, overly restrictive, inappropriate environments.
Red patch on tip of snout	Rostral lesion/abrasion	Stress. Persistent attempts to push against, crawl up, dig under or round the transparent barriers of their enclosure

*Derived from reviewed literature (1, 8, 44, 57, 79, 81, 115, 116). Where any of the above signs are observed, veterinary opinion should be obtained*.

#### Cause/problem keys

The tables (Tables 7A–F) provide a summary of relatively common behavioral signs of welfare concerns and their potential associated problematic causes. Where a “Behavioral sign” is observed (e.g., “Lethargy”), refer to the “Cause/Problem” column (e.g., “1, 6”), and then proceed to the “Cause/problem keys” below the relevant table to find the potential cause or problem.

## Scoring the establishment using the checklist and the “star”/traffic light system

**Figure 2:**
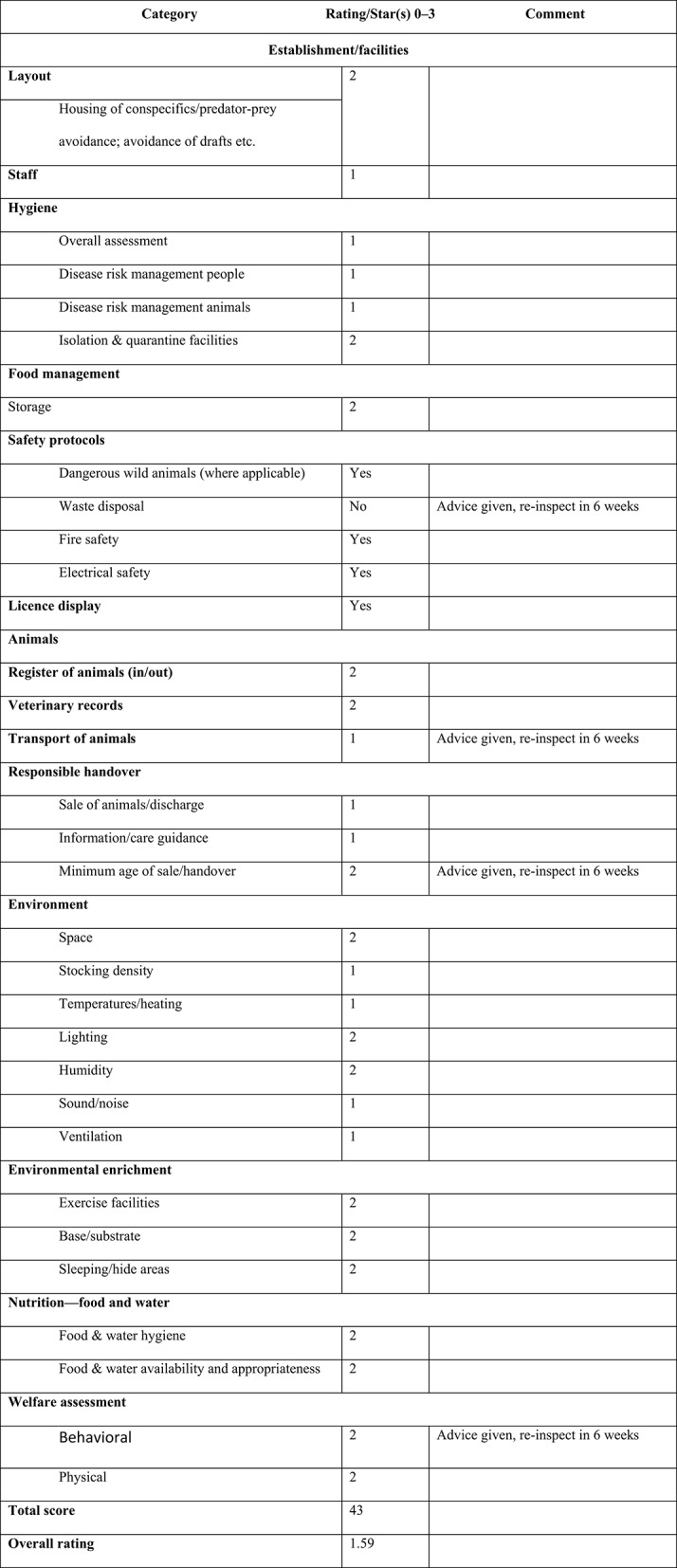
Sample overall performance rating chart for animal establishment (refer to Appendix 1 for blank template).

Inspection may apply a “star” or a “traffic light” scoring system to both general and individual sections of an establishment based on 14 categories and 27 subcategories to deliver the relevant “checklist” scores (see Figure 2).

The scoring system is intended to be user-friendly, and the guidance Tables and Figures allow for objective assessment. While primarily intended for use by local authority inspectors, the scoring system may also be of use to establishment managers who may wish to periodically “self-assess” using the same criteria. Figure 2 provides an example checklist that may be used to score the performance of an animal establishment.

Inspectors may award a score based on the following assessment: 0 = Poor/failed; 1 = Adequate; 2 = Good; 3 = Exemplary.

Calculating points for a “score” rating.

1. There are 27 scoring categories in the checklist
2. Each category may be awarded a score of 0–3
3. When all categories have been scored, add together all scores
4. In the sample completed checklist (Figure 2) the total points scored = 43
5. Divide the total scored by 27 to find the average score, so 43/27 = 1.59
6. Scores over 0.5 are rounded up, and scores under 0.5 are rounded down
7. In the sample, the score 1.59 can be rounded up to the next whole point or “2”
8. 2 points = 2 “stars”
9. The establishment may be awarded a “2-star” “Good” rating.

An inspector may choose to isolate and score more specific aspects of the establishment's animal facilities, or opt to score on a more generalized basis. If certain parts of the establishment's protocols or provisions are markedly better than others, isolated scoring will help emphasize which areas/enclosures are maybe “exemplary” and which are “poor” and need improvement. In the sample checklist, we have adopted the generalized approach. However, where an inspector elects to isolate and score on a more specific basis, they should use the following method for calculating points and star ratings, For example:

- There are 30 individual animal enclosures in an establishment
- 10 receive 1 point each, 15 receive 2 points each, and 5 receive 3 points each
- Therefore, total points scored for animal facilities = 55 points
- Divide the total scored by 30 to find the average score, so 55/30 = 1.83
- Scores over 0.5 are rounded up, and scores under 0.5 are rounded down
- In the sample, the score 1.83 can be rounded up to the next whole point “2”
- 2 points = 2 “stars”
- The establishment may be awarded a “2-star” “Good” rating for animal facilities.

According to the nature of any relevant concern resulting low scores, the inspector should consider what action is appropriate to remedy a situation. Such action may involve providing guidance regarding improvement and set a time limit for compliance, or may involve immediate decisive intervention as may be available.

As a condition of establishment licensing, authorities should consider stipulating the species that may be offered for sale within the formal authority's, or the available inspector's, own competence level (Appendix 1 provides a blank template for use).

### Points, stars, and traffic lights

**Figure 3:**
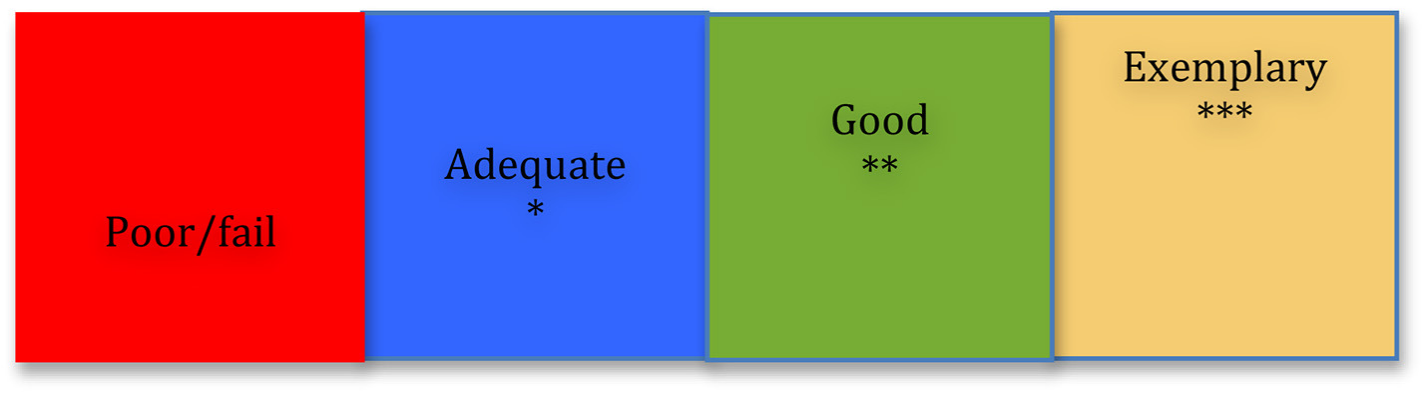
Sample traffic-lights system for establishment standard-setting.

The use of a points and stars system also enables establishments to be rated according to a quick-view color standard for public assurance. Establishments obtaining a zero score involve poor facilities that constitute “poor/fail” and in a traffic lights system (Figure 3) would be assigned a “red light.” Whilst an establishment receiving a red light would unlikely wish to display that flag, the absence of any flag would indicate poor performance. Establishments obtaining scores of 1 or 2 leading to “blue/adequate” or “green/good” flags may use these as indicators of their respectability, which despite differences in level demonstrates their operations as distinct from poor. Establishments obtaining 3 scores leading to “gold/exemplary” level facilities provide both indication of best practice and also act as inspiration for other establishments to raise their standards. It is possible that an inspector may wish to assign a 3 score/3 star/gold level rating to a single exemplary aspect or facility of an establishment, rather than the entire establishment.

## Conclusions

Animal welfare as well as human health and safety considerations conjoin across a diversity of animal establishments. Overseeing these establishments, however regulated, requires onsite inspection by suitably qualified, impartial, and competent personnel. Standardization of modern inspection methodology provides a “safety net” level for management protocols aimed at delivering reasonable protections both for animals and people. Mandatory minimum standards of husbandry and inspection may be augmented by incentivization of establishments to voluntarily further raise operating conditions to achieve recognized distinctions, which also offer elevated public assurance. We believe that this is the first impartial resource that meets the “three-asset” target for providing guidance regarding animal husbandry for diverse establishments, inspection protocols, and the provision of a relevant assessment tool to aid the guidance.

## Author contributions

All authors contributed equally to the concept and design of the paper. CW prepared the first draft. CW, MJ, PA, AP, and CS provided additional research and writing input to the final manuscript.

### Conflict of interest statement

CW, CS, and MJ have acted as professional consultants to the Animal Protection Agency. The Animal Protection Agency contributed financially to the literature search and review by CW and CS, whilst having no design, research, data collection, analysis, interpretation, writing, decision-making, or other directional role in this report. The remaining authors declare that the research was conducted in the absence of any commercial or financial relationships that could be construed as a potential conflict of interest.

## Figures and Tables

**Appendix 1:**
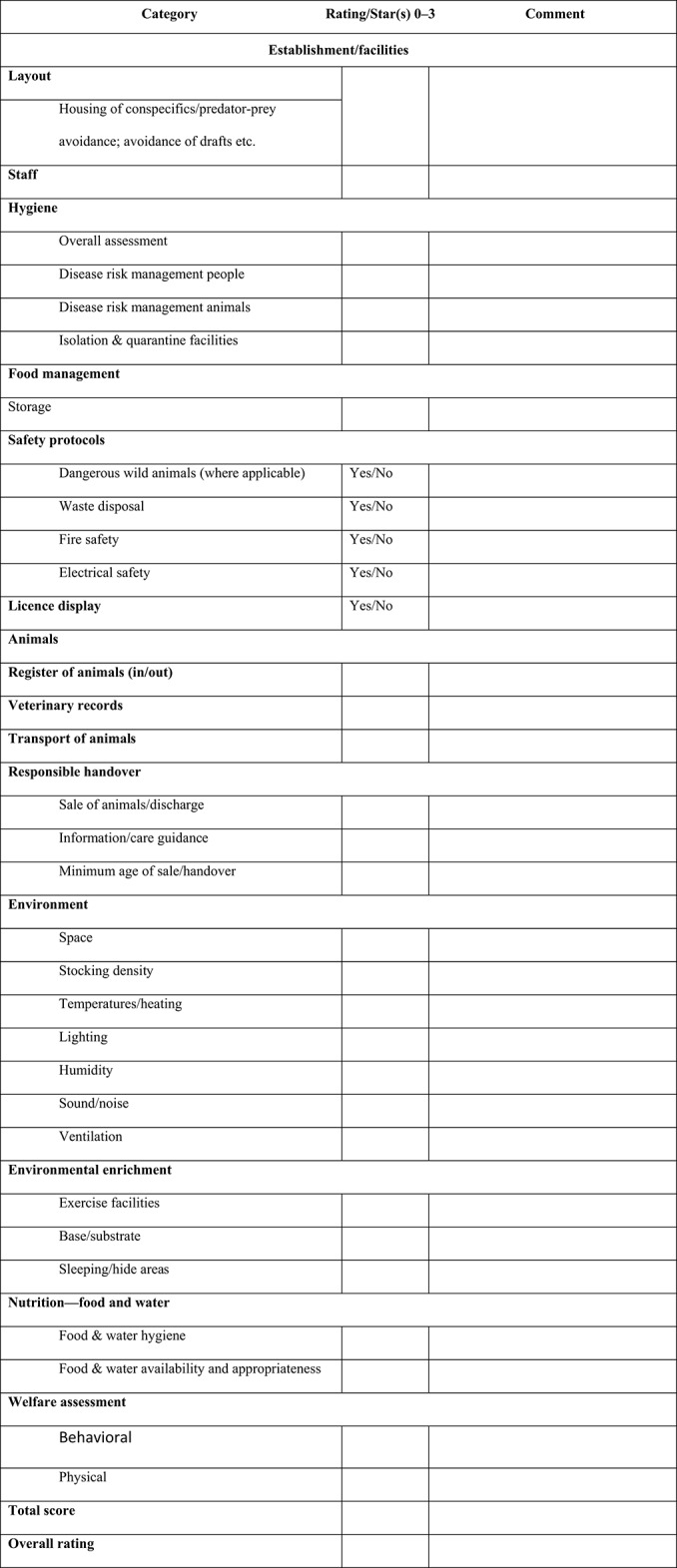
Template: overall performance rating chart for animal establishment.
